# Men deny more than they believe about climate change on Twitter (X)

**DOI:** 10.1371/journal.pone.0303007

**Published:** 2025-02-13

**Authors:** Mudit Kumar Singh

**Affiliations:** 1 Social Science Research Institute, Duke University, Durham, NC, United States of America; 2 G L Bajaj Institute of Technology and Management, Greater Noida, Uttar Pradesh, India; Roma Tre University: Universita degli Studi Roma Tre, ITALY

## Abstract

Climate change and twitter have been in scholarly and academic attention for study of human behaviour expressed on the popular social media platform. The sentiment of the tweets has been the subject of previous studies, and the most recent study used Twitter texts to examine seven aspects of climate change: denier/believer stance, sentiment, aggressiveness, temperature, gender, subjects and disasters, and their relationships. Amid the big pictures across these vital variables, we know very little about the extent to which the comparative gendered differences in views exist in the climate denier and believer groups shaping the climate change discussion. Using the large scale global twitter data from the past 13 years, this paper has examined the differences in the views of deniers and believers on climate change in comparison to the people neutral to climate change. Based on the expression on twitter, results of a sound multinomial regression model of this study indicates a globally strong climate denier stance of men.

## Introduction

Social media plays crucial roles in this debate and contradiction. Platforms like Twitter can function as a discussion forum, a soft power tool, and a vehicle for transnational advocacy and climate action [1]. Of all the social media networks, Twitter is a particularly great resource for disaster risk management research. Twitter was launched in October 2006 and quickly became the largest microblogging service [2]. The policymakers often use social media, and Twitter in particular, to frame the debate and to showcase particular achievements [3,4]. Twitter serves as a powerful platform for disseminating real-time information about climate change. Researchers, activists, and organizations share updates, scientific findings, and urgent alerts related to environmental issues [5]. For instance, during natural disasters like hurricanes, wildfires, or floods, Twitter becomes a vital channel for emergency notifications, evacuation instructions, and relief efforts [6]. The immediacy of tweets allows people worldwide to stay informed and take necessary precautions. Twitter played a crucial role in spreading awareness during extreme weather events [7], emphasizing the need for climate action [8].

Twitter facilitates global dialogue on climate change. Hashtags like #ClimateAction, #ClimateCrisis, and #ClimateJustice trend regularly, sparking conversations among diverse audiences. Scientists share their research, policymakers discuss policy implications, and activists mobilize support for environmental causes. The platform amplifies voices that advocate for sustainable practices, renewable energy, and conservation. By connecting people across borders, Twitter fosters a sense of collective responsibility [9]. As a result, Twitter is an important part of the context in which many actors in the global climate change regime develop political views, contest climate norms, but also spread misinformation and disinformation. The growing interest among scholars in various countries is focused on how the general public utilizes social media to participate in discussions related to climate change and potential solutions. Twitter data can also play a crucial role in analyzing how opinions on climate change and climate policy are formed among both citizens and elites. By studying how political communication or endorsements from individuals can impact public opinions on these issues, researchers can gain valuable insights. Future studies on public opinion could further explore these analyses using Twitter data, investigating whether specific framings of climate risks, such as climate-induced displacement, can influence public opinion through social media content.

These research also shed light on the potential advantages and disadvantages of different framings of climate change, as they can impact climate governance through their influence on the general public. While many studies have progressively used sentiment analysis through texts and visualization of tweets about climate change worldwide, causality and probability of individual attributes (e.g. gender) causing such tweets are further areas to explore about climate change tweets.

This work delivers a key contribution especially by introducing application of a causal analytical model to all the research effort that has been made in the field of climate change and Twitter. Past works have studied the sentiment of the tweets and the most recent work explored seven dimensions of climate change via Twitter texts namely, stance, sentiment, aggressiveness, temperature, gender, topics and disasters, and their interactions. This work has focused on magnitude of difference between stance (denier, believer and neutral) climate change stances while exploring the causal linkage between gender and stance along with the other key components.

### Literature review

Big Data analytics techniques have been widely used to evaluate environmental management and sustainability problems in order to uncover hidden trends and patterns in public opinions. Climate change denial often hinges on disputing temperature data, with some arguing that Earth’s climate has historically fluctuated naturally [10].

Climate change is a pressing global issue with significant implications for society, and social media platforms have become instrumental in facilitating discussions around it [11]. Scholars have recognized the role of social media in shaping public perceptions, attitudes, and behaviors towards climate change [12]. Platforms like Twitter, Facebook, and Instagram serve as spaces where individuals share information, express opinions, and engage in dialogue about climate-related topics [13,14].

Studies have highlighted the potential of social media to amplify climate change discourse and foster public engagement [15–17]. For instance, research by [18] found that Twitter discussions on climate change often include a diverse range of voices, from scientists to activists to policymakers. Furthermore, social media enables rapid dissemination of climate-related news and information, allowing users to stay informed and connected to developments in real-time [14].

However, scholars have also identified challenges associated with climate change discourse on social media, including the spread of misinformation, polarization of opinions, and echo chambers [19]. This is further advanced by other scholars who have explored polarization in digital media environments such as climate change debates on Twitter [20–26].

Skeptics may question the significance of current temperature increases or attribute them to cyclic patterns unrelated to human activity. Cherry-picked data or misinterpretation of climate models is common tactics to cast doubt [27]. Such denial obstructs efforts to address climate change, despite overwhelming scientific consensus on its reality and human causation [28]. Recognizing and countering denialism is crucial for implementing effective climate policies and mitigating its far-reaching impacts.

Despite methodological and skeptical challenges, social media platforms remain critical spaces for engaging diverse audiences in discussions about climate change and mobilizing collective action towards mitigation and adaptation efforts [14].

A majority of the data used in these analyses comes from the microblogging service such as Twitter [29]. Several aspects of climate change through human opinions have been studied over the past years by researchers. The early studies of climate change on Twitter by [30,31] considered whether important events affect the discussion, and identified three groups of human stance towards climate change, namely supportive, unsupportive and neutral. [32] reported correlation between human sentiment and both hot and cold temperatures, underlining the importance of temperature as an important variable in climate change sentiments. Skeptics may question the significance of current temperature increases or attribute them to cyclic patterns unrelated to human activity. Cherry-picked data or misinterpretation of climate models is common tactics to cast doubt [27]. Such denial obstructs efforts to address climate change, despite overwhelming scientific consensus on its reality and human causation [28]. Recognizing and countering denialism is crucial for implementing effective climate policies and mitigating its far-reaching impacts.

Resource overconsumption and human intervention profoundly influence the climate change debate, often exacerbating contradictions between deniers and believers [33]. Overconsumption, particularly of fossil fuels, accelerates greenhouse gas emissions, fueling climate change. Deniers, often backed by vested interests, may downplay these impacts to protect economic interests [34]. In contrast, believers emphasize the urgent need for sustainable resource management and emissions reductions to mitigate climate change’s adverse effects. However, societal dependence on resource-intensive lifestyles and industries complicates efforts to enact meaningful change, perpetuating the contradiction between recognizing climate change’s reality and addressing its root causes. The differences in opinions of climate change believer and denier groups exist across many variables including gender. Gender is reported to play a role in attitude towards climate change. While both genders use very similar language, female tweeters had a convinced attitude and male tweeters presented a skeptical stance [35] towards impact of climate change. Finally, another aspect of concern for many researchers is the topics discussed in climate change related tweets [36].

Twitter serves as a crucial platform for political discussions on climate change, and the data derived from it is frequently utilized in the analysis of non-state climate action and public opinion on social media.

Therefore, there exists significant potential to enhance our understanding of how actors within the climate governance complex engage on Twitter, interact with the general public, and are influenced by these interactions. A deeper comprehension of climate debates on Twitter can contribute to climate governance research and advance theories on the impact of social media, including norm diffusion, opinion leadership, and the formation of citizen and elite opinions, on climate governance.

### Data and methods

I have used twitter data available on Kaggle [37]. It is claimed to be one of the most extensive dataset available on Twitter that relates to human attitudes and climate change. With over 13 years of temporal coverage, over 15 million tweets geographically dispersed worldwide. Each tweet is analyzed across seven informational dimensions: geolocation, user gender, aggression, climate change attitude and sentiment, deviations from historical temperatures, topic modeling, and data on environmental disaster occurrences. The data used by [37] suggests that these dimensions are the result of testing and analyzing numerous cutting-edge supervised and unsupervised machine learning algorithms and techniques.

The previous studies have heavily relied on text analysis and descriptive techniques of countrywide variation of sentiments and tweets about climate change and have produced useful information. These studies have used text analysis approaches to uncover the pattern and variations of climate change discussion across the globe. In order to have significant insights across the vital opposite groups (believer and denier) of climate change, a robust analytical model is required. Given the three stances, suitable model choices are ordered logit or multinomial. Respecting the opinions of individuals, it will be highly appropriate to not to rank the groups i.e. to make any order of three groups. In this case, it is useful to go for the multinomial model that does not consider any order between the outcome categories. Therefore, in this way, adding to the contributions discussed in the literature review section, I will use multinomial logistic regression model to assess the relationship between the dependent variable (stances: believer, neutral or denier) and the key independent variables. Where the independent variables are temperature, gas emission, impact of overconsumption, tweet language (aggressiveness) and gender. These variables are given in the dataset and are categorized based on the expression of individuals on the twitter. Thus, these categorized expressions will help understanding the likelihood of individuals taking their stance (believer, or denier, or neutral) for the climate change. While all the aforementioned independent variables are important, for the purpose of this paper, I have maintained a focus on gender during detailed interpretation of result.

To assess the relationship between the qualitative outcome variable (stances of climate change by individuals via tweets), I have used the multinomial logistic regression represented by the following equation.


$$\ln(\pi_{j}/\pi_{j})=A_{j}+B_{j}X$$

The logistic model is a generalization of the binary outcome of standard logistic regression comparing each category of the outcome (climate believer and denier in this case) to a referent category (neutral). The equation assumes, there are J total categories of the outcome, indexed by the subscript j, and the number of comparisons is then J– 1. The equation is written in terms of the logit of the outcome, which is a comparison of a particular category to the referent category, both denoted as *π*_*j*_ in the equation. The j subscript on both the intercept, *A*_*i*_, and slope, *B*_*j*_, indicate that there is an intercept and a slope for the comparison of each category which is a generalized case of binary logistic model.

The predicted probability for each predictor can be computed by generalizing the above equation for standard logistic, using the following equation.


$$\pi_{j}=1/(1+\sum e^{A_{j}+B_{j}X})$$

The model is used by earlier studies on climate change in the context of agriculture [38] and is the best choice for this data as the outcome has three unordered categories- believer, neutral and denier and there are sufficient numbers of cases across the categories over such a large dataset (millions of tweets). Conceptually, the model selection is also based on the assumption that no one is considered superior in holding their stance towards climate change and hence the stances cannot be ordered.

Before running the model, I omitted the year 2006 as it contained only four valid observations after dropping the bank cells and also dropped the ‘undefined’ category of gender. After the cleaning, the values in data were left with the year 2006 to 2017. A descriptive summary of variables used in the analysis is given in Table 1 ahead. I have used RStudio software and *nnet* package [39] to conduct multinomial regression.

**Table 1:** Displays the descriptive of key variables summary of the categorical variables used in the model.

Variables	Categories	Frequency	% of N
Stance	Neutral	343751	32.78%
	Believer	595818	56.82%
	Denier	109006	10.40%
Gender	Female	310124	29.58%
	Male	738451	70.42%
Topics	Ideological Positions on Global Warming	74106	7.07%
	Impact of Resource Overconsumption	64975	6.20%
	Importance of Human Intervention	320802	30.59%
	Seriousness of Gas Emissions	139207	13.28%
	Weather Extremes	449485	42.87%
Aggression	Aggressive	322527	30.76%
	not aggressive	726048	69.24%
	Total number of cases		N = 1048575

The table indicates low number of cases in two categories (ideological positioning on global warming and impact of resource over consumption) of variable ‘Topics’. In all the other variables, at least 10% cases are present within a variable. The % distribution of cases across the variable categories suggest sufficient number of cases to run the multinomial model with no blank cells as those were dropped from the data in cleaning process. The first category of each variable was held as referent to interpret the results. The model converged in 20 iterations and the significant chi-square test (33936, P<0.00) shows the statistical significant of the model [40]. The results are discussed in the following section.

## Results

Table 2 shows the results of the model. While the sign of the coefficients tells the nature of comparative differences or similarity of opinions between the deniers and believer groups; the log-odds interpretation and relative-risk ratio calculation further helps understanding the size of differences in opinions of the two groups across these variables.

**Table 2:** Results of multinomial regression.

	(Intercept)	temp	Impact of Resource Overconsumption	Importance of Human Intervention	Seriousness of Gas Emissions	Weather Extremes	not aggressive	male
believer	0.44(0.01)	0.02(0.00)	0.16(0.01)	0.95(0.01)	0.55(0.01)	-0.3(0.01)	-0.05(0.00)	-0.14(0.00)
denier	-0.64(0.01)	-0.04(0.00)	-0.85(0.02)	-0.92(0.01)	0.05(0.01)	-0.32(0.01)	-0.51(0.01)	0.27(0.01)
neutral	Reference category							

(N = 1048576, Chi square = 33936, p = 0.000).

The signs are vital in this model output as the opposite signs are indication of significant difference in comparative stances of climate change believer and denier groups in comparison to the referent category of neutral. The variables showing similar sign of coefficients may differ in magnitude but will move in the same direction i.e. the (log) odds will either increase (for positive sign) or decrease (for negative sign) but will not show a comparative difference of opinions. While the signs of coefficients of “seriousness of gas emissions”, “weather extremes”, “aggression” remains same, the other variables show difference in stances and these include the key variable of interest “gender” for this paper.

Put simply, in the terms of log odds, the variable gender can be interpreted as follows. One unit increase in gender will reduce the log odds of being a climate believer vs neutral by 0.14 and one unit increase in gender will increase the log odds of being a denier vs neutral by 0.27.

I have further taken exponential values of all the coefficients to calculate the associated relative risk ratio for the interpretation from a different perspective. The relative risk ratio for a one-unit increase in the variable gender is .87 for being a believer vs. neutral whereas, the same goes as high as 1.31 for being a denier vs. neutral.

Similarly, for other variables, we find that relative risk ratio for a unit increase in temperature is 1.02 for believer and 0.96 for denier in comparison to neutral group. Likewise, these values for remaining variable are 1.17 and 0.43 (resource over consumption), 2.59 and 0.40 (human intervention), 1.73 and 1.05 (seriousness of gas emission), 0.74 and 0.73 (weather extremes), 0.95 and 0.60 (non-aggressive individuals). Thus, except weather extremes, we see moderate to high differences in relative risk of being in the believer and denial groups in the data set. This suggests the posts about weather extremes are produced almost equally by the climate change denial and believer groups across the globe but they have fundamental differences about the cause of the same. We see a consistent trend of high risk ratio among believing climate change groups for all the variables that are found positively related to climate change by the studies applying text analysis using the same data set [41].

The key concern of the paper is the gender effect in the expression on twitter about climate change. Is the similar gender affect detected by other scholars in case of climate change? Based on the review of other scientific studies, the findings on gender extend the findings of [35] that indicates the males take skeptical stance for climate change impact. In a recent empirical study by [42] in the USA, it is found that climate change concerns are affect gender though, economic outlook moderate the effect of gender. To extend these findings, in a relevant context, a global survey conducted by [43] asserts that both men and women are less concerned about climate change in wealthier countries than in poorer countries but that men’s concern will decline more rapidly, creating a gender gap in climate concern. The study sample is not as wide as the twitter data set used in this study so probably the results are not in conformity with the results of this study in entirety but underscore the importance of gendered attitude in fight against climate change.

### Limitations

The study is not without limitation. One of the most widely accepted critique of twitter data is the identity of tweeting individuals. We are not 100% certain about the correct information about a bot, or an individual on twitter. So, we cannot be sure if the gender data is correctly provided to the twitter by the users. However, given the large scale data context, we assume this would not create a big difference on results. Therefore, we may hope that the conclusions are generalizable. The data only contained binary gender. So, the results are limited to male and female only. The other limitation was not using the location data as it would generate a long list of countries. This would add complexity in model that would create distraction and distortion in the interpretation of the results of the existing multinomial egression being used and hence, using the location data is beyond the scope of this study.

## Conclusion and future research

The paper presents a useful multinomial model that can be used to assess the probability of differences in groups holding an opinion on the social media data like Twitter (now known as X). A silver lining of the analysis is that all the groups are serious about climate change gas emission. However, there is difference in the opinions among the groups with a significant variation across the two gender categories of the two groups. The findings are useful for policy makers and climate change activists as it reveals the sceptical stance of men that means use of awareness and communication materials targeted to sensitize and improve understanding of men about the climate change to meet the global target of achieving net zero. From the limitation discussed above, using the location data, future research may focus on country or region specific variations that scholars can use to generate useful insights about the variables shaping climate change discussion on twitter. Adding to the recent studies discussed in the result section, the other vital question that researchers may further explore why do men take skeptical stance on climate change belief in various regions and countries? Additionally, based on the discussion on moderating effect by scholars, it is important to understand what are the variables that moderate the gendered discussion about climate change? This will help developing a broader understanding whether the finding is true only for Twitter social media or do we have any variation in the statement based on location, social media platform or population not using social media in real world.
